# Sense of Agency during Encoding Predicts Subjective Reliving

**DOI:** 10.1523/ENEURO.0256-24.2024

**Published:** 2024-10-10

**Authors:** Nathalie Heidi Meyer, Baptiste Gauthier, Jevita Potheegadoo, Juliette Boscheron, Elizabeth Franc, Florian Lance, Olaf Blanke

**Affiliations:** ^1^Laboratory of Cognitive Neuroscience, Neuro-X Institute, Faculty of Life Sciences, Ecole Polytechnique Fédérale de Lausanne, Geneva 1202, Switzerland; ^2^Clinical Research Unit, Neuchâtel Hospital Network, Neuchâtel 2000, Switzerland; ^3^Department of Clinical Neurosciences, University Hospital Geneva, Geneva 1205, Switzerland

**Keywords:** autonoetic consciousness, episodic memory, self-consciousness; sense of agency, virtual reality

## Abstract

Autonoetic consciousness (ANC), the ability to re-experience personal past events links episodic memory and self-consciousness by bridging awareness of oneself in a past event (i.e., during its encoding) with awareness of oneself in the present (i.e., during the reliving of a past event). Recent neuroscience research revealed a bodily form of self-consciousness, including the sense of agency (SoA) and the sense of body ownership (SoO) that are based on the integration of multisensory bodily inputs and motor signals. However, the relation between SoA and/or SoO with ANC is not known. Here, we used immersive virtual reality technology and motion tracking and investigated the potential association of SoA/SoO with ANC. For this, we exposed participants to different levels of visuomotor and perspectival congruency, known to modulate SoA and SoO, during the encoding of virtual scenes and collected ANC ratings 1 week after the encoding session. In a total of 74 healthy participants, we successfully induced systematic changes in SoA and SoO during encoding and found that ANC depended on the level of SoA experienced during encoding. Moreover, ANC was positively associated with SoA, but only for the scene encoded with preserved visuomotor and perspectival congruency, and such SoA–ANC coupling was absent for SoO and control questions. Collectively, these data provide behavioral evidence in a novel paradigm that links a key subjective component of bodily self-consciousness during encoding, SoA, to the subjective reliving of those encoded events from one's past, ANC.

## Significance statement

We provide behavioral evidence showing that the sense of agency during the encoding of immersive virtual scenes as modulated by visuomotor and perspectival congruency modulates autonoetic consciousness, thereby linking a key component of the bodily self during encoding with the subjective reliving of the encoded events.

## Introduction

Autonoetic consciousness (ANC) is an important feature of episodic memory and is defined as the re-experiencing of an event that a person has experienced in the past. Introduced by Endel Tulving (Tulving, 1985; M. A. Wheeler et al., 1997), the notion of ANC centers on subjective aspects of re-experiencing during the retrieval of sensory, emotional, and personal aspects tied to the specific spatiotemporal context of a past event. ANC involves an awareness of oneself in the past (during the encoding of the event) and an awareness of oneself in the present (during the reliving the encoded event; Levine, 2004; Winocur and Moscovitch, 2011; Prebble et al., 2013; Markowitsch and Staniloiu, 2014), being placed at the intersection of self and memory (Tulving, 1985; Schacter et al., 2003; Piolino et al., 2006; Markowitsch and Staniloiu, 2011, 2014). This autonoetic ability of self-projection in the past (and future) has been argued to enable continuity of the self across time (Levine, 2004; Piolino et al., 2009; Vandekerckhove, 2009; Prebble et al., 2013), being part of the remembered self or extended self.

Concerning self and memory, ANC has also been linked to a different form of self based on bodily perception and sensorimotor processes (Klein et al., 2004, 2011; Klein, 2013). This bodily self (Ehrsson, 2007, 2012; Blanke and Metzinger, 2009; De Vignemont, 2011; Blanke et al., 2015) includes the sense of agency (SoA), defined as the feeling of being in control of one's body and its actions (David, 2010; Kannape and Blanke, 2013; Haggard, 2017), and the sense of body ownership (SoO), defined as the feeling that the body belongs to oneself (Blanke and Metzinger, 2009; De Vignemont, 2011; Blanke et al., 2015). Klein and colleagues (Klein et al., 2004) emphasized the co-occurrence of episodic memory deficits (including ANC) with SoA deficits in patients with schizophrenia and with SoO deficits in a neurological patient, proposing that ANC and episodic memory deficits could be associated with alterations in SoA and/or SoO (Klein et al., 2004; Klein and Nichols, 2012; Klein, 2013). However, such coupling of SoA and SoO with ANC has neither been investigated experimentally in healthy participants nor confirmed in subsequent clinical studies.

Recent research developed several methods to modulate the SoA and SoO experimentally, by using virtual reality (VR) and exposing participants to different visuotactile or visuomotor stimulations (Fourneret and Jeannerod, 1998; Frank et al., 2001; Kannape et al., 2010; Kannape and Blanke, 2013; Salomon et al., 2022) or by changing the perspective from where the body of the participant and the virtual scene is seen (first-person perspective, 1PP, or third-person perspective, 3PP; Ehrsson, 2007, 2012; Brugger and Lenggenhager, 2014; Blanke et al., 2015). However, only a few studies investigated the association between bodily self-consciousness and ANC, and most of them did not measure the subjective state linked with the experimental manipulation of bodily self-consciousness (Bréchet et al., 2019, 2020; Gauthier et al., 2020). To date, only one study explored whether the experimental manipulation of the subjective SoO during encoding modulates the later subjective reliving of the encoded events (ANC). Iriye and Ehrsson (2022) observed that an altered SoO through visuotactile stimulation applied during the encoding period was associated with a decrease in some aspects of ANC, such as emotional intensity. However, the studies that did measure subjective ratings did not systematically investigate the link between these ratings during encoding and the later acquired subjective ANC (Bergouignan et al., 2014; Iriye and Ehrsson, 2022).

Here, we directly investigated potential links between the bodily self and ANC by exposing participants to different levels of visuomotor and perspectival congruency to change the SoA and SoO during the encoding of virtual scenes. We then tested the potential effects of the SoA/SoO modulation on ANC, 1 week after the encoding session. We gauged ANC for the encoded virtual events by using questions developed for testing ANC for real-life events in a total of 74 healthy participants.

## Materials and Methods

We recruited a total of 82 participants; 26 participants (7 male; mean age 23 ± 3.4 years) for Experiment 1, 29 participants (11 male, 3 gender-nonconforming, mean age 24 ± 3.4 years) for Experiment 2, and 27 participants (10 male, mean age 27 ± 3.5) for Experiment 3. All participants were right-handed (Flinders Handedness Survey; FLANDERS, Nicholls et al., 2013) and reported no history of neurological or psychiatric disorder and no drug consumption in the 48 h preceding the experiment. We determined our sample size based on previous studies with a similar experimental design, expecting comparable effect sizes (Bréchet et al., 2019, 2020; Gauthier et al., 2020; Iriye and Ehrsson, 2022). These studies recruited between 16 and 33 participants. Additionally, to ensure that we could maintain a minimum of 24 participants for the randomized condition-scene matching across participants, we accounted for the possibility of participant exclusions. The study was approved by the local ethical committee (Cantonal Ethical Committee of Geneva: 2015-00092, and Vaud and Valais: 2016-02541) and respected the declaration of Helsinki. All participants provided written informed consent and received financial compensation for their participation. Behavioral and fMRI data from all participants from these 3 experiments have been partly reported previously, concerning recognition memory performed 1 h after encoding (Meyer et al., 2024). The present data concern a separate data acquisition about ANC that was collected in a different session 1 week after encoding, separately for each of the three experiments (see methods below).

### Material and technical setup

In Experiments 1 and 3, participants were lying down in a mock magnetic resonance (MR) scanner wearing a head-mounted display (Oculus Rift S; refreshing rate, 80 Hz; resolution, 1,280 × 1,440 per eye, 660 ppi) and holding custom response devices in their hands (two hand-held tennis balls with integrated buttons and reflective 6 degree-of-freedom motion trackers) to simultaneously record participants’ answers and track participants arm movement using three motion-tracking cameras (Qualisys Oqus 500+ m cameras with 180 Hz, 4 megapixel resolution). Experiment 2 was similar, but participants were lying in an MR scanner; therefore, they were wearing MRI-compatible goggles allowing stereoscopic rendering at 60 Hz with a diagonal field of view of 60° (Visual System HD, NordicNeuroLab) similar to a previous study (Gauthier et al., 2021). Instead of three motion-tracking cameras, the MR scanner contained six motion-tracking cameras to track participants’ body movements.

The experiments consisted of three sessions: an incidental encoding session, a recognition task 1 h after the encoding, and a questionnaire to quantify ANC. For the scope of this paper, we will not discuss the results of the recognition task and will focus on the ANC assessment.

### Encoding session

**Figure 1.**
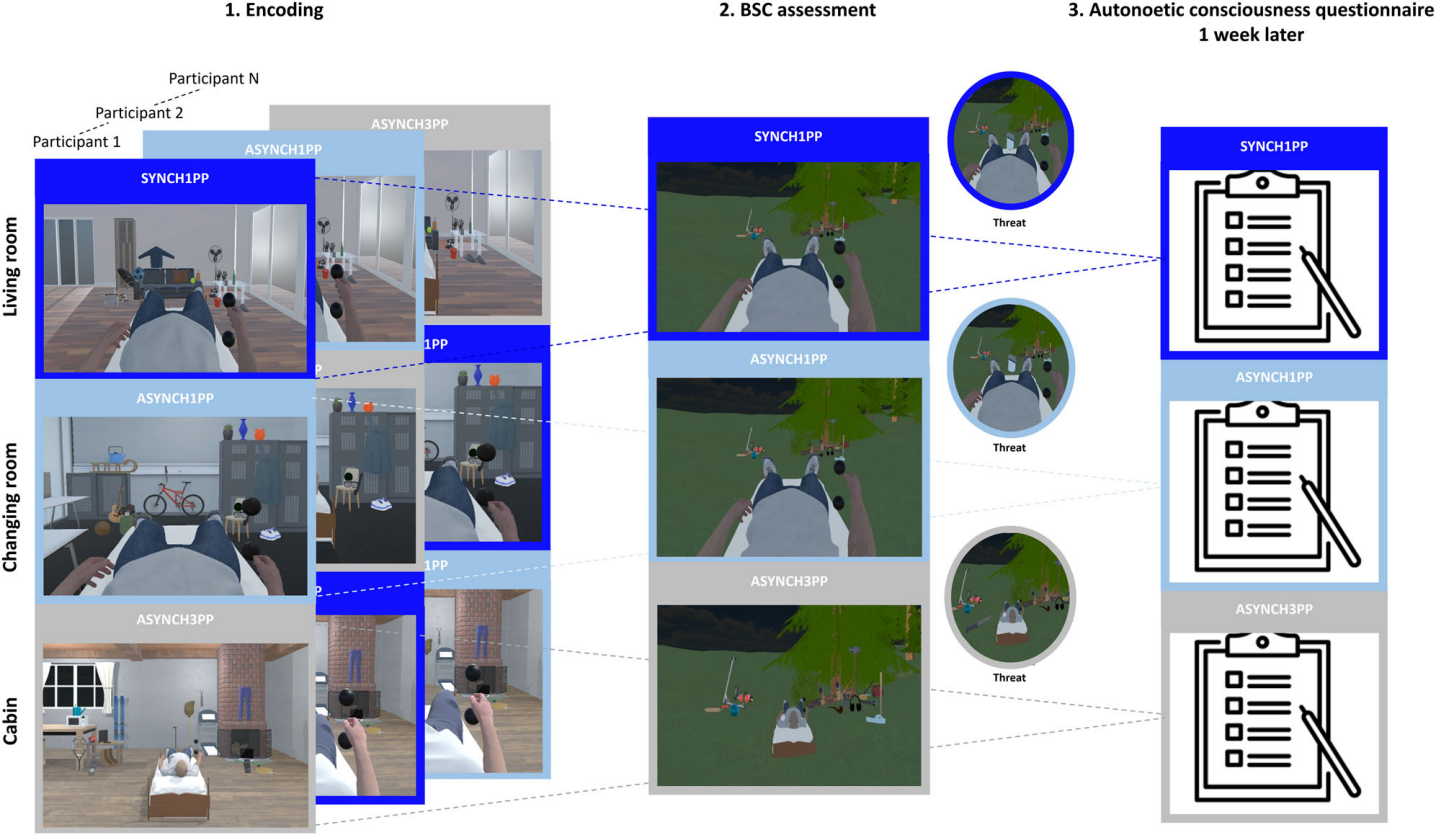
Experimental design: Participants encoded three scenes corresponding to three different conditions (left panel). One scene was encoded under preserved visuomotor and perspectival congruency (SYNCH1PP, dark blue), while another was encoded under visuomotor incongruency with preserved perspective (ASYNCH1PP, light blue). The last scene was encoded under visuomotor and perspectival incongruency (ASYNCH3PP, gray). The association between scene and condition was pseudorandomized between participants. After the encoding session, participants were immersed in a fourth outdoor scene (middle panel) in which they experienced the three conditions (SYNCH1PP, ASYNCH1PP, and ASYNCH3PP). After performing the same movements as during encoding, they observed a virtual knife arriving in the virtual avatar (middle panel, colored circle). They were asked to rate their level of sense of agency (SoA) and sense of ownership (SoO) and whether they were afraid to be hurt by the knife (implicit SoO, threat ratings) and answered some control questions to evaluate their bodily self-consciousness. One week after the encoding session, participants were called back to answer an autonoetic consciousness questionnaire for each scene (and therefore each encoding condition).

Participants were instructed to move their right arm between two virtual black spheres while observing an avatar during the encoding of three indoor virtual scenes containing 18 objects each. Each scene was presented for 30 s and associated with a specific modulation of visuomotor and perspectival congruency: (1) no modulation (SYNCH1PP, preserved bodily self-consciousness), where participants observed the avatar at the first-person perspective with the avatar arm moving synchronously with the participants’ movement; (2) visuomotor incongruency (ASYNCH1PP, light manipulation of bodily self-consciousness), where participants observed the avatar at the first-person perspective but the movement were asynchronous (fluctuating delay between 800 and 1,000 s); and (3) visuomotor and perspectival incongruency, in which the avatar was shifted in front of participant's view at the third-person perspective additionally to the asynchrony (ASYNCH3PP, strong manipulation of bodily self-consciousness). The association between the scene and the conditions was pseudorandomized between participants (Fig. 1). Each scene was presented four times for each experimental condition with an intertrial of 5 s to avoid potential carry-over effects from one condition to another. Before the experiment started, participants performed a familiarization procedure, during which they were immersed in an outdoor virtual scene and asked to perform right arm movement while observing an avatar moving first in synchrony and then asynchronously with respect to their movement.

### Assessment of SoA and SoO

Immediately after the encoding, participants were presented with a fourth outdoor scene in which we assessed the bodily subjective experience related to the bodily self-consciousness state. They were given the same instruction as during the encoding session and observed an avatar in SYNCH1PP, ASYNCH1PP, and ASYNCH3PP (each presented twice for 35 s, with a randomized order, Fig. 1). After the first 30 s, a virtual knife appeared on the scene and moved into the virtual body for 5 s. After which, the participants had to rate one statement about their SoA (“I felt that I was controlling the virtual body”), SoO (“I felt that the virtual body was mine”), response to the virtual threat (“I was afraid to be hurt by the knife”), and two control items, unrelated to SoA and SoO (“I felt that I had more than three bodies”; “I felt that the trees were my body”). The addition of a threat allowed us to gauge SoO with a second more implicit question (“I was afraid to be hurt by the knife”), to avoid potential floor effects for the main SoO question (“I felt that the virtual body was mine”; i.e., potential floor effect, because the visual aspects of the avatar did not match with those of the participants such as color, size, clothes, etc; Armel and Ramachandran, 2003; Kilteni et al., 2012a; Ma and Hommel, 2015; Gonzalez-Franco and Lanier, 2017; Moon et al., 2022). For each statement, a cursor was programmed to move continuously between the two extreme points of the agreement scale (from 0, totally disagree, to 1, totally agree, with increments of 0.001) at a constant speed. Participants needed to stop the cursor at their desired position by pressing the left button and then confirm their response with a right button press. Before confirming, participants could retry as many times as needed, using the left button to adjust their agreement level until they were satisfied with their choice.

### Autonoetic consciousness assessment

One week after the encoding, ANC of participants was assessed for each of the three encoded VR scenes, separately during a phone call (Fig. 1). For each scene encoded (and therefore each condition), participants were asked to answer a series of questions taken from the “memory characteristics questionnaire” (MCQ, Johnson et al., 1988) and the “episodic autobiographical memory interview” (EAMI Part B, Irish et al., 2008, 2011). Table 1 describes each of the 28 items of the questionnaire. The participants were asked to recall what happened during the experiment they performed the week before, while they were in VR. To ensure this, they were asked to focus on one specific scene at a time (and thus a specific condition). They were cued with the name of the respective scene from a total of three scenes (“living room,” “cabin,” “changing room”). They were asked to briefly describe the scene before answering the questionnaire to make sure that the questions would capture the specific condition associated with the remembered scene.

**Table 1.** Autonoetic consciousness (ANC) questionnaire

Statement	Item	Scale	Reference
My memory for this event is	E1	1–7 dim/clear	MCQ
My memory for this event involves visual details	E2	1–7 little/a lot	MCQ
My memory for this event involves sound	E3	1–7 little/a lot	MCQ
My memory for this event involves smell	E4	1–7 little/a lot	MCQ
My memory for this event involves touch	E5	1–7 little/a lot	MCQ
My memory for this event involves taste	E6	1–7 little/a lot	MCQ
My memory for this event is	E7	1–7 sketchy/very detailed	MCQ
My memory for the location where the event takes place is	E8	1–7 vague/distinct	MCQ
Relative spatial arrangement of objects in my memory for the event is	E9	1–7 vague/distinct	MCQ
The relative spatial arrangement of people in my memory for the event is	E10	1–7 vague/distinct	MCQ
My memory for the time when the event took place is	E11	1–7 vague/distinct	MCQ
The overall tone of the memory is	E12	Negative/neutral/positive	MCQ
The intensity of the overall tone of the memory is	E13	1–7 weak/strong	MCQ
In this event, I was	E14	A spectator/a participant	MCQ
I remember how I felt at the time when the event took place	E15	1–7 not at all/definitely	MCQ
I remember what I thought at the time	E16	1–7 not at all/definitely	MCQ
When I think about or tell this memory, I feel like I relive it as it happened	E17	1–7 not at all/definitely	MCQ
When I remember the event, I see myself entirely in the scene as if I was watching a movie	E18	1–7 not at all/definitely	MCQ
I remember the event through my own eyes as during the event	E19	1–7 not at all/definitely	MCQ
I remember the movements and gestures I made with my body at the time of the event	E20	1–7 vague/distinct	Experiment-specific
When you recall this event how would you describe it in terms of vividness? This can apply to the richness of sights, sounds, smells, tastes, touch, and any movements you may have made	EAMI1	1–7 very vivid/very vague 1–7 very vague/very vivid	EAMI
When you recall this event, are you viewing the scene through your “own eyes” or can you see yourself in the memory from a third-person perspective?	EAMI2	Own eyes/mixture/third person/something different/no imagery	EAMI
When you picture this event, do you visualize it as a continuous video that plays with break, moving video clips with some breaks, or one moving image, or is it more like a set of snapshots with no movement, or something else?	EAMI3	1–7 one smooth video/video clips with breaks/one moving image/snapshot in sequence/one static snapshot/hazy image/no image No image/Hazy image/one static snapshot/snapshot in sequence/one moving image/video clips with breaks/one smooth video	EAMI
How often would you estimate you have thought about this memory since it first occurred?	EAMI4	1–4 Frequently/occasionally/rarely/never Never/rarely/occasionally/frequently	EAMI
How often would you estimate you have spoken about this memory since it first occurred?	EAMI5	1–4 Frequently/occasionally/rarely/never Never/rarely/occasionally/frequently	EAMI
When you think about this event now, do you re-experience any of the emotions you originally felt at the time? To what extent are you re-experiencing this emotion as a percentage?	EAMI6	0/25/50/75/100%	EAMI
Would you say you are reliving this memory or looking back on it?	EAMI7	Reliving/looking back	EAMI
To what extent are you re-experiencing this memory as a percentage?	EAMI8	0/25/50/75/100%	EAMI

ANC questionnaire. The scale from the original questionnaire is indicated in black, and the new scale is indicated in green. ANC, autonoetic consciousness; EAMI, episodic autobiographical memory interview; MCQ, memory characteristics questionnaire.

### Behavioral data analysis

Behavioral analysis was carried out using R (R Core Team, 2022), R Studio (RStudio, 2022), and Python. Linear mixed models were computed in R using the package *lme4* and *lmerTest*. In total, we removed eight participants (three from Experiment 1, two from Experiment 2, and three from Experiment 3) because of technical issues or high ratings in the control questions. Thus, we performed the analysis on 74 participants in total.

### Sense of agency and sense of body ownership

To quantify how the experimental conditions during encoding modulated SoA and SoO, we applied linear mixed models to explain SoA and SoO, respectively, as dependent variables, the conditions as fixed factors (three levels: SYNCH1PP, ASYNCH1PP, and ASYNCH3PP), the experiment as fixed covariate (three levels: Experiment 1, Experiment 2, and Experiment 3) and the participants as random factor. We averaged the ratings between trials to obtain one score for each rating (SoA, SoO, threat, control). For the response to the virtual threat and for the control ratings, we applied the same analysis (for the analysis of the control items, we averaged the two control questions).

### Autonoetic consciousness score

We reversed the scale of three questions extracted from the EAMI questionnaire (EAMI4, EAMI1, EAMI4, EAMI5; see Table 1 for the items detail) to have higher ratings corresponding to stronger reliving (the original EAMI questionnaire associate the lowest ratings, 1, as strong vividness and 7 a slow vividness for example). Original scaling is depicted in black and reversed scaling in green in Table 1.

We first described the overall ANC for the different virtual scenes by computing the number of occurrences of extreme ratings for items of the questionnaire taken from the EAMI questionnaire [based on the approach by Irish et al. (2011)]. Additionally, we computed the average score for different categories of the MCQ (clarity, items E1, E2, E17, and E7 from Table 1; context, items E9 and E10; nonvisual sensory, items E3, E4, and E6; thoughts/feelings, items E15 and E16; intensity of feeling, item E13) based on a previous study (Hashtroudi et al., 1990).

To further investigate the role of ANC with SoA and/or SoO, we then normalized the ratings for each item by dividing the answered value by the maximum ratings possible for each item. We computed an ANC score for each participant and for each condition by summing the normalized ratings of each participant. Thus, we obtained one global score of ANC for each participant and each condition. The maximum ANC score is 28, as there are 28 items in the questionnaire.

### Autonoetic consciousness and sense of agency

To investigate whether the SoA and/or SoO at encoding modulated ANC collected 1 week later, we compared two linear mixed models: The first explained the ANC score (dependent variable) with the conditions (Model 0) the second explained the ANC score (dependent variable) with the conditions and interaction with the SoA score (Model 1). For each model, the experiment was added as a covariate (fixed factor with three levels: Experiment 1, Experiment 2, Experiment 3), and the participants were added as random factors. We selected the model with the lowest AIC that also passed the *χ*^2^ test. To further investigate whether the ANC score was better explained with the other bodily self-consciousness ratings, we performed the same model comparison for SoO and threat ratings.

Model 0: ANC_score_ ∼ conditions + experiment + random effect of participants

Model 1: ANC_score_ ∼ conditions*SoA + experiment + random effect of participants

Once we selected the best model, we computed similar models for SoO, threat, and control ratings.

ANC_score_ ∼ conditions*SoO + experiment + random effect of participants

ANC_score_ ∼ conditions*threat + experiment + random effect of participants

ANC_score_ ∼ conditions*control (average of both control questions) + experiment + random effect of participants

In case of significant interaction, post hoc correlations were performed separately for each condition between the dependent variable and the covariate. The condition SYNCH1PP was used as the reference condition for each model. In the case of the model explaining ANC with SoA, we also looked at the interaction between ASYNCH1PP and ASYNCH3PP to investigate whether the interaction found was due to the change of perspective. In that specific case, we changed the reference condition to ASYNCH1PP.

### Code availability

The behavioral data and analysis code will be accessible through the OSF platform at the conclusion of the review process (https://osf.io/5f3eb/?view_only=dcd4c644c4d846a3b666e734b17e56dd).

## Results

### Higher SoA under visuomotor and perspectival congruency

Our main interest was to investigate whether different levels of the SoA and/or SoO, as modulated by visuomotor and perspectival congruency during encoding, affected ANC ratings recorded 1 week later. For this, we combined the data from all three experiments (Experiments 1, 2, and 3) leading to a total of 74 participants. We first report the data on SoA and SoO. We expected higher ANC for the scene encoded under preserved visuomotor and perspectival congruency and, additionally, explored the effect of both SoA and SoO on ANC collected 1 week later, without a specific hypothesis about a differential role of SoA versus SoO in their impact of ANC.

**Figure 2.**
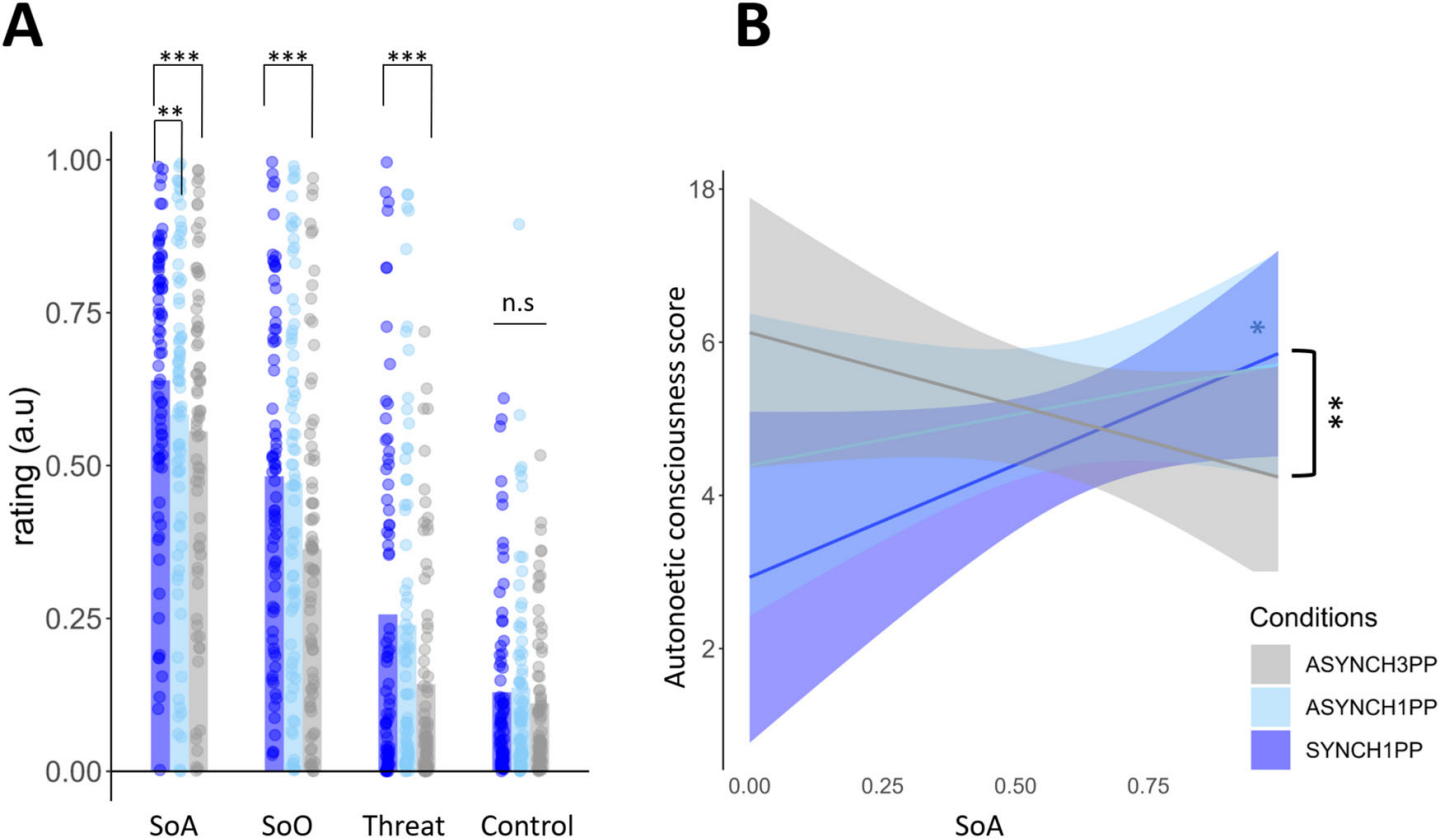
Impact of visuomotor and perspectival congruency on bodily self-consciousness and autonoetic consciousness. ***A***, Sense of agency (SoA) was higher under visuomotor and perspectival congruency (SYNCH1PP, dark blue) compared with the two manipulated conditions (ASYNCH1PP, light blue; ASYNCH3PP, gray) as tested with a linear mixed model with SoA as dependent variable and conditions as fixed factor (*N* = 74). The statistical analysis supporting these results is displayed as a table in Extended Data Figures 2-1 and 2-4. ***B***, There was a significant interaction between visuomotor and perspectival congruency (SYNCH1PP, dark blue) and visuomotor and perspectival mismatch (ASYNCH3PP, gray) as tested with a linear mixed model, with ANC score as dependent variable, and interaction between conditions and SoA as independent variable (*N* = 74). SoA was positively correlated with ANC score only under visuomotor and perspectival congruency. The statistical analysis supporting these results is displayed as a table in Extended Data Figures 2-5 and 2-8. * and ** indicate significance level with *p*-value <0.05 and 0.001, respectively. ANC score was computed as the sum of the normalized ratings of all the ANC questionnaire items for each condition and each participant. ANC, autonoetic consciousness; SoA, sense of agency; SoO, sense of body ownership.

During encoding, participants had a higher SoA in the condition with preserved visuomotor and perspectival congruency (SYNCH1PP, Fig. 2*A*) compared with the other two conditions (ASYNCH1PP and ASYNCH3PP; SYNCH1PP compared with ASYNCH1PP: estimate = −0.059, *t* = −3, *p* = 0.003. SYNCH1PP compared with ASYNCH3PP: estimate = −0.08, *t* = −4.28, *p* < 0.001. ASYNCH1PP compared with ASYNCH3PP: estimate = −0.025, *t* = −1.26, *p* = 0.21). We found similar results for SoO, for which participants had significantly higher ratings under preserved visuomotor conditions compared with the condition with strongest visuomotor and perspectival mismatch (SYNCH1PP compared with ASYNCH3PP: estimate = −0.12, *t* = −2.14, *p* = 0.036; the SoO did not differ significantly between SYNCH1PP vs ASYNCH1PP: estimate = −0.003, *t* = −0.12, *p* = 0.9). There were no significant differences between conditions for the control ratings (SYNCH1PP compared with ASYNCH1PP: estimate = 0.007, *t* = 0.729, *p* = 0.47. SYNCH1PP compared with ASYNCH3PP: estimate = −0.018, *t* = −2.578, *p* = 0.08).

10.1523/ENEURO.0256-24.2024.f2-1 Figure 2-1 Sense of agency. Sense of agency ∼ Conditions + Experiment + random(Participants). Download Figure 2-1, DOCX file.

10.1523/ENEURO.0256-24.2024.f2-2 Figure 2-2 Sense of ownership. Sense of ownership ∼ Conditions + Experiment + random(Participants). Download Figure 2-2, DOCX file.

10.1523/ENEURO.0256-24.2024.f2-3 Figure 2-3 Threat. Threat ∼ Conditions + Experiment + random(Participants). Download Figure 2-3, DOCX file.

10.1523/ENEURO.0256-24.2024.f2-4 Figure 2-4 Control.Control ∼ Conditions + Experiment + random(Participants). Download Figure 2-4, DOCX file.

10.1523/ENEURO.0256-24.2024.f2-5 Figure 2-5 Autonoetic consciousness explained by SoA and Conditions. ANC ∼ Conditions * SoA + Experiment + random(Participants). Download Figure 2-5, DOCX file.

10.1523/ENEURO.0256-24.2024.f2-6 Figure 2-6 Autonoetic consciousness explained by Control and Conditions. ANC ∼ Conditions * Control + Experiment + random(Participants). Download Figure 2-6, DOCX file.

10.1523/ENEURO.0256-24.2024.f2-7 Figure 2-7 Autonoetic consciousness explained by Threat and Conditions. ANC ∼ Conditions * Threat + Experiment + random(Participants). Download Figure 2-7, DOCX file.

10.1523/ENEURO.0256-24.2024.f2-8 Figure 2-8 Autonoetic consciousness explained by SoO and Conditions. ANC ∼ Conditions * Ownership + Experiment + random(Participants). Download Figure 2-8, DOCX file.

Additional analysis revealed that the SoA ratings did not significantly differ between the three experiments (Extended Data Fig. 2-1). SoO and control ratings for participants of Experiment 3 were overall higher than Experiments 1 and 2 (Extended Data Figs. 2-2, 2-4). However, post hoc analysis separating Experiment 3 from Experiments 1 and 2 confirmed the predicted finding that the SoO was significantly higher in the condition with preserved visuomotor and perspectival congruency compared with the strongest mismatch (ASYNCH3PP). This analysis also confirmed the absence of significant differences between conditions for the control ratings. Detailed results for SoO, SoA, threat, and control questions are depicted in Extended Data Figures 2-1 and 2-4. Together, these results demonstrate that we successfully modulated the level of bodily self-consciousness (SoA, SoO) by exposing a large group of participants to different levels of visuomotor and perspectival incongruency while they encoded scenes in VR.

### Autonoetic consciousness is related to SoA, when the events were encoded under visuomotor and perspectival congruency

We next analyzed whether visuomotor and perspectival congruency during encoding modulated ANC recorded 1 week later and especially whether the subjective changes in bodily self-consciousness across conditions (SoA, SoO) improved the model. Concerning the SoA, we found that the participants’ ANC scores were better explained by the model combining the experimental conditions with the SoA scores (Model 1) compared with the model explaining the ANC scores only with the experimental conditions alone (Model 0, *χ*^2^ test; Model 1 was significantly better than Model 0, AIC_model0 _= 1,087, AIC_model1 _= 1,084, df = 3, *p* = 0.024). Moreover, we found that depending on the encoding condition, the SoA was related to the ANC score as tested with a linear mixed model: There was a significant interaction between SYNCH1PP and ASYNCH3PP (with SoA estimate = −4.34, *t* = −2.99, *p* = 0.003, Fig. 2*B*, Extended Data Fig. 2-5), but not when comparing SYNCH1PP with ASYNCH1PP (estimate = −2.27, *t* = −1.58, *p* = 0.12). To investigate whether this interaction may have been due to collinearity in our linear mixed model, we calculated the variance inflation factor (VIF) for the model without interaction (ANC ∼ conditions + SoA + experiment + random effect of participant) and found a VIF of 1 for each covariate (conditions, 1.06; SoA, 1.08; Experiment 1.01). We also quantified the VIF for the model with the interactions (SoA, 1.25; Experiment 1.01; conditions, 6.1; conditions/SoA, 5.9). As we found a VIF lower than 10 but higher than 5, our model indicates moderate collinearity and thus does not reach a level that would compromise the validity of our findings. Post hoc power analysis revealed that the interaction had a statistical power of 71% (confidence intervals were between 61.07 and 79.94). Further post hoc analysis applied separately on each condition revealed a significant correlation between SoA and the ANC scores only in the encoding condition with preserved visuomotor and perspectival congruency (SYNCH1PP, *r* = 0.28, *t* = 2.42, df = 71, *p* = 0.018), which was absent in the two other conditions (ASYNCH1PP, *r* = 0.12, *t* = 1.05, df = 71, *p* = 0.3; ASYNCH3PP, *r* = −0.16, *t* = −1.36, df = 71, *p* = 0.19). Finally, to better understand whether the significant interaction was driven by a change of perspective, we applied the same model to compare ASYNCH1PP with ASYNCH3PP. Even though both conditions only differed in perspective, there was no significant interaction (estimate = −2.05, *t* = −1.55, *p* = 0.12), suggesting that the effect is not driven by a change of perspective.

The results differed for SoO, threat, and control items. Thus, we did not observe that the participants’ ANC scores were better explained by the model combining the experimental conditions with SoO or threat scores nor by the control ratings (Model 1, AIC_SoO _= 1,091, AIC_Threat _= 1,089, AIC_Control _= 1,092; Model 0, AIC = 1,087), suggesting that the additional parameters added in the model are valuable only in the case of SoA. Moreover, there was no significant interaction between the SoO and the ANC score as tested with a linear mixed model (SYNCH1PP compared with ASYNCH1PP: estimate = −0.42, *t* = −0.32, *p* = 0.75. SYNCH1PP compared with ASYNCH3PP: estimate = −1.86, *t* = −1.326, *p* = 0.19. ASYNCH1PP compared with ASYNCH3PP: estimate = −1.44, *t* = −1.1, *p* = 0.27). There was also no significant interaction between conditions when applying the same analysis to threat ratings or control ratings (Extended Data Figs. 2-6, 2-8).

### Autonoetic consciousness for virtual scenes has ratings comparable to real events encoded in the laboratory

Concerning the overall ANC scores, we report that participants had an average ANC score of 53% (mean, 15.01; SD, 3.37) of the maximally possible ANC score (100% corresponding to a score of 28). Seventy-five percent of subjects reported reliving the virtual scene as if seeing it from their own eyes in our study. Seventeen percent of our participants had a high overall re-experiencing, and 14% reported a strong emotional re-experiencing. Finally, 13% of the participants were able to relive the virtual events (see Table 2 for more details and discussion for comparison with previous work).

**Table 2.** Extreme ratings in the EAMI part of the ANC questionnaire

	item	Overall % of subjects with these answers
Reliving	EAMI7	13%
Vividness (very vivid/vivid)	EAMI1	26%
Vividness (very vague/very vague)	EAMI1	5%
Viewer perspective (own eyes)	EAMI1	75%
Viewer perspective (third person)	EAMI1	11%
Continuity of mental imagery (smooth video)	EAMI3	8%
Continuity of mental imagery (no image)	EAMI3	1%
Emotional re-experiencing (75–100%)	EAMI6	14%
Emotional re-experiencing (0%)	EAMI6	21%
Covert rehearsal (very often)	EAMI4	8%
Covert rehearsal (never)	EAMI4	1%
Overt rehearsal (very often)	EAMI5	0%
Overt rehearsal (never)	EAMI5	44%
Overall re-experiencing (75–100%)	EAMI8	17%
Overall re-experiencing (0%)	EAMI8	14%

Extreme ratings in the EAMI part of the ANC questionnaire. The column item refers to the item detailed in Table 1 used to compute the occurrence of the extreme ratings in this table.

We used a similar approach to compare the ratings on items taken from the MCQ questionnaire. When grouping our results in similar categories as done by previous authors (Hashtroudi et al., 1990), we found that our participants indicated high values for vividness (5.2; max ratings, 6) and reported being able to recollect their thoughts (5.6; max ratings, 6), while they gave lower values for the recollection of nonvisual sensory factors (1.4; max ratings, 6; see Table 3 for more details and discussion for comparison with previous work).

**Table 3.** Global scores between categories proposed by Hashtroudi et al. (1990)

Category	Items	Average
Visual details and vividness	E1, E2, E7, E17	5.2
Spatial arrangement of people and objects	E9, E10	5.3
Nonvisual sensory factor	E3, E4, E6	1.4
Thought and feeling factors	E16, E15	5.6
Intensity of feelings	E13	1.7

Global scores between categories of the memory characteristics questionnaire (MCQ) proposed by Hashtroudi et al. (1990). The column items details which items from Table 1 were grouped together to form the average score reported in the column average.

## Discussion

We investigated the role of the bodily self in ANC by systematically changing the SoA and SoO during the encoding of virtual scenes. For this, we exposed participants to different levels of visuomotor and perspectival congruency during the encoding of objects in three different scenes and collected ANC ratings 1 week after the encoding session. Using immersive VR and motion tracking, we successfully induced (1) systematic changes in the SoA and SoO during encoding and found that (2) ANC collected 1 week after the encoding session depended on the level of SoA experienced during encoding. Moreover, (3) the reliving of the encoded scene (ANC) was positively associated with the SoA, but only for the scene encoded with preserved visuomotor and perspectival congruency, which is in the SYNCH1PP condition, when participants encoded the scene while seeing congruent movements from their 1PP. Such SoA–ANC coupling was (4) only found for SoA and was absent for SoO and control questions. Collectively, these data provide behavioral evidence in a novel paradigm that links a key subjective component of bodily self-consciousness during encoding, SoA, to the subjective reliving of those encoded events from one's past, ANC.

### Changes of SoA and SoO during the encoding of virtual scenes

Our results show that we successfully induced changes in the bodily self (Fourneret and Jeannerod, 1998; Frank et al., 2001; Blanke and Metzinger, 2009; De Vignemont, 2011; Blanke et al., 2015; Haggard, 2017) by exposing our participants to different visuomotor and perspectival conditions that disrupted sensorimotor processes in real-time, while our participants encoded virtual scenes. We note that these changes were achieved using immersive VR combined with motion tracking, in line with previous work using VR to test SoA and SoO (Kilteni et al., 2012b; Padilla-Castañeda et al., 2014; Pan and de Hamilton, 2018; Slater et al., 2019; Freude et al., 2020). As expected, our participants reported higher SoA and SoO ratings under preserved visuomotor and perspectival congruency (in the SYNCH1PP condition) and lower SoA and SoO ratings when they were exposed to a visuomotor and perspectival mismatch, as reported in previous research (Ehrsson, 2007; Lenggenhager et al., 2007; Kannape and Blanke, 2013; Bergouignan et al., 2014). This was fundamental to allow us to study the main question of this study: how changes in SoA and/or SoO during encoding impact ANC, collected a week later.

### ANC depends on SoA experienced during encoding a week earlier

We employed immersive VR to simulate experimental conditions that approximate scenes and objects in real life by testing three different complex indoor scenes. Critically, each scene differed in the level of visuomotor and perspectival congruency, counterbalanced across participants and allowing us to test later SoA–ANC coupling. In each condition, participants were immersed and observed a particular 3D scene and objects as well as their avatar while they moved their right hand. ANC for the scenes was tested 1 week after the encoding session by a questionnaire consisting of items created to measure ANC for real-life events (Johnson et al., 1988; Irish et al., 2008, 2011). Of note, during reliving, no virtual scene, avatar, or body view was shown to participants (i.e., we prompted them orally by the name of each scene, e.g., the word “cabin”).

Our model comparison showed that including the SoA experienced at encoding improved the linear mixed model to explain ANC compared with an analysis that only included the effect of the different experimental conditions (i.e., the effect of visuomotor and perspectival congruency). This links a key subjective component of bodily self-consciousness during encoding, the SoA, to the subjective reliving of these events (ANC), encoded 1 week earlier. This was further supported by our finding that such SoA–ANC coupling differed for the three experimental conditions. Thus, ANC was positively related to the SoA only under preserved visuomotor and perspectival congruency, meaning that stronger SoA at encoding was coupled with stronger reliving, indexed by higher ANC ratings collected a week after the encoding. Moreover, this relationship was not present for scenes encoded under visuomotor and perspectival mismatch. Thus, although the overall ANC values were similar across all conditions, only the scene encoded with preserved SoA showed SoA–ANC coupling. This suggests that there was no difference in ANC between our conditions per se but that ANC was retrieved by a different strategy or process since it was coupled with SoA only under visuomotor and perspectival congruency, but not under visuomotor and perspectival mismatch. Although some studies have shown that ANC was stronger under preserved bodily self-consciousness (Bergouignan et al., 2014; Iriye and Ehrsson, 2022), these studies used fewer and more selective questions to capture ANC, which were selectively targeting aspects of the chosen experimental task. Our approach was different since we gauged ANC by questions that are traditionally used to measure ANC for autobiographical events. Since our virtual scenes were not as rich as an autobiographical event, the difference in ratings between conditions might have been too low to capture the effect of conditions.

The positive coupling between SoA and ANC under visuomotor and perspectival congruency suggests that the SoA trace is either better integrated when the scene was encoded under preserved visuomotor and perspectival congruency or that the events with higher SoA are better retrieved. An interesting question would be to understand whether the observed SoA–ANC coupling depends on the level of SoA experienced at a given moment or whether it is a specific trait depending on the general SoA level of a given subject. Our results tend to suggest that the coupling is more likely to depend on the SoA level experienced during the encoding of a specific event as we found the same level of ANC for scene encoded under disrupted visuomotor and perspectival congruency, independent from the SoA level. Future studies should directly investigate this question. Critically, the coupling with ANC was absent for control ratings. Related work has shown that objects encoded before an emotionally salient event were better retained (Dunsmoor et al., 2015; Patil et al., 2017). Moreover, Rimmele and colleagues (Rimmele et al., 2011, 2012) showed that the emotional valence of an event (e.g., negative) at encoding can strengthen ANC during later reliving. We argue that the SoA may impact ANC in a comparable way but is likely mediated via different neural networks (via sensorimotor agency networks rather than emotional networks coupled with memory and ANC networks). Several studies have highlighted the central role of the hippocampus in the reactivation of sensory cortical areas involved in encoding during the later recall of an event (Nadel and Moscovitch, 1997; Wheeler et al., 2000; Moscovitch et al., 2016; Sekeres et al., 2017, 2018). However, none of these studies discussed the place of bodily self-consciousness in the hippocampal–neocortical axis. Our results indicate that the hippocampus may also index the reactivation of brain regions involved in bodily self-consciousness (Park and Blanke, 2019) during the re-experience of an event, especially when this event was encoded under preserved visuomotor and perspectival congruency. These findings provide empirical support for earlier clinical proposals that SoA is an important component of ANC (Klein et al., 2004; Klein and Nichols, 2012). Critically, our results underline that SoA–ANC coupling is especially present when events are encoded in conditions of preserved visuomotor and perspectival congruency. This could mean that patients with SoA deficits (such as patients with schizophrenia; Corcoran and Frith, 2003; Hauser et al., 2011; Maeda et al., 2012; Potheegadoo et al., 2013) or patients with ANC deficits (as in amnesia) could benefit from new memory rehabilitation therapies focusing on enhancing SoA during encoding and during retrieval in order to increase memory performance and ANC.

Although SoA and SoO were both systematically modulated across our conditions, we did not observe SoO–ANC coupling. We note that our experimental sensorimotor conditions aimed at modulating SoA, for which we observed a significant reduction in both conditions with visuomotor and perspectival mismatch. Concerning SoO, we observed the same reduction of ratings in the strong visuomotor and perspectival mismatch condition (ASYNCH3PP) for both our explicit SoO ratings and our implicit measure of SoO (threat ratings), supporting the successful experimentally-induced decrease of bodily self-consciousness across our conditions with visuomotor and perspectival mismatch. Although Iriye and Ehrsson (2022) showed that SoO, as manipulated by visuotactile stimulation during encoding, was positively related to increased vividness and emotional intensity in ANC ratings collected 1 week later, several reasons may account for the difference between these and our results. First, the threat to the virtual body observed before the different ratings of bodily self-consciousness might have affected the SoO of participants and therefore hindered the potential SoO–ANC coupling not observed in our study. In addition, there are differences in stimulation applied during encoding between both studies. Thus, the latter authors applied visuotactile stimulation during encoding, known to induce changes in SoO (but not SoA; Lenggenhager et al., 2007; Blanke and Metzinger, 2009; Noel et al., 2015; Iriye and Ehrsson, 2022; Moon et al., 2024), while our study applied a visuomotor manipulation known to modulate SoA (Kannape and Blanke, 2013; Padilla-Castañeda et al., 2014). Another difference is that the former study applied stimulation to passive observers, whereas we used an active paradigm during which participants carried out continuous movements. Finally, our experimental design included an additional change in perspective and used immersive VR while that of a previous study (Iriye and Ehrsson, 2022) used prerecorded 3D video, projected to a 1PP view, rendering direct comparison between both studies difficult. Future work is needed to systematically compare the role of SoA and SoO, across different experimental conditions. Our data also extend earlier important work showing ANC changes when adopting a 3PP versus a 1PP at encoding (Bergouignan et al., 2014; Iriye and St Jacques, 2018, 2021). Using a different active visuomotor paradigm, we did not find any ANC differences depending on perspective (comparing conditions ASYNCH1PP vs ASYNCH3PP). However, given the marked differences in experimental paradigms in these different studies, any direct comparison is difficult, and more systemic studies are needed. Nevertheless, all studies converge in demonstrating that different aspects of bodily self-consciousness (SoA, SoO, perspective; Blanke, 2012; Blanke et al., 2015) may impact ANC, as related work has shown for episodic memory (Bréchet et al., 2019, 2020; Iriye and Ehrsson, 2022; Penaud et al., 2022).

### Autonoetic consciousness in virtual reality is similar to autonoetic consciousness for events generated in the laboratory

One of the main difficulties when measuring real-life autobiographical memories is that the experimenter has no access to the encoding moment of these events and can therefore neither account nor control for the many potential confounds that may affect retrieval (Smith, 2019). To assess ANC, researchers “ask participants to recall events from a particular time frame (even though) there is often no experimental control over characteristics of the events recalled such as their importance, emotional content, prior rehearsal, or the quality of recollective experience” (Levine, 2004). Another difficulty with ANC is its subjective nature. Accordingly, the study of ANC (and autobiographical episodic memory) under experimental laboratory settings as done for other aspects of memory is desirable. However, this has proven very difficult or impossible (i.e., creating events with the same level of autobiographical relevance as a real-life autobiographical event). The present ANC ratings obtained for different complex indoor scenes using immersive VR to simulate complex realistic scenes and events are comparable to ANC ratings reported for events staged inside the laboratory (Hashtroudi et al., 1990), as well as events outside the laboratory (Irish et al., 2011). Concerning ANC ratings for events staged inside the laboratory, Hashtroudi et al. (1990) asked participants to come to the laboratory and carry out specific tasks (e.g., pack a picnic basket, and visit an auditorium) and then tested ANC using the memory characteristics questionnaire (Johnson et al., 1988) 1 day later. Grouping the questions into different categories, they obtained a vividness score of 6 (their Fig. 2) for healthy participants (present study, 5.2), a score of 6 for spatial arrangement (present study, 5.3), a nonvisual sensory score of 2 (present study, 1.4), and a thought score of 4.5 (present study, 5.6). These data show that ANC for immersive virtual scenes can generate similar ANC ratings, compared with events encoded in the laboratory. Critically, immersive VR has the advantage of being fully reproducible and fully controlled (Pan and de Hamilton, 2018) and avoids the need to have trained actors during encoding procedures (Albert et al., 2024). Moreover, our ANC ratings also compare with those obtained for real-life events outside the laboratory. Thus, we observed that 75% of the participants in our study reported reliving the virtual events through their own eyes and that 17% have an overall strong re-experiencing. Both values are in the range of what has been reported for real-life events outside the laboratory. Irish and colleagues (Irish et al., 2011) measured ANC for autobiographical events and found that 62% of participants felt viewing events with their own eyes and that 7% noted a strong global re-experiencing. Some subcomponents of the questionnaire were lower in our study, compared with the study by Irish et al. (13% of our participants reported reliving the events in which they were in the virtual scenes compared with 33% in the former study), but this is not surprising given the comparison between rich personally relevant events from real-life events with more controlled and less personal laboratory events in VR. Overall, these results indicate that our virtual scenario allows the generation of controlled and complex immersive scenes that can be employed to investigate ANC and episodic memory (Bréchet et al., 2019, 2020; Gauthier et al., 2020; Meyer et al., 2024).

### Limitations

This study was not aimed at disentangling distinct effects of SoA versus SoO in ANC but rather provided empirical evidence to show that ANC depends on sensorimotor bodily processes during encoding and its related subjective state of bodily self-consciousness. It would have been interesting to also test the effect of conditions in which only the SoO (and not the SoA) was altered; however, this would have significantly increased the already long experiment duration. Accordingly, we employed a gradient, from SYNCH1PP to ASYNCH1PP to ASYNCH3PP, for which we expected a gradual manipulation of the SoA. This was sufficient to test our main research questions, while minimizing experiment duration, tiredness of participants, and task difficulty. Future studies should be designed to disentangle potentially distinct effects of subcomponent of bodily self-consciousness (i.e., SoA vs SoO) and their potential differential impact on ANC. We also note that the present study combined three different experiments, with different instruction and VR systems. It is likely that these differences added some variability in the data. However, as we did not find any effect of the experiment in our mixed model analysis, this variability did not impact the key findings of this study. Despite encouraging results, this study did not aim to measure nor generate autobiographical events. ANC strength, as measured for lab-based virtual scenes, was still lower than what has been reported for autobiographical events from real life (Irish et al., 2011). Future studies need to consider the tradeoff between using the standard approach (asking people to rate personally relevant real-life events from a larger time period) and the present immersive VR approach that is more controlled but does not assess major autobiographical life events. Finally, both SoA and ANC are self-reported measures and could have been subject to suggestibility bias (Donaldson and Grant-Vallone, 2002) in the present study. The following reasons make it unlikely that this is the case. First, we note that the positive relationship between SoA and ANC was found only under visuomotor and perspectival congruency, but not for conditions with visuomotor and perspectival mismatch (ASYNCH1PP and ASYNCH3PP). Second, ANC ratings were acquired a week after the encoding session, and third, our participants were not aware of the aims of our study (except in Experiment 3). Fourth, we found no ANC effects or coupling for SoO or control questions, making it overall very unlikely that SoA and ANC ratings were affected similarly by such potential biases.

## Synthesis

Reviewing Editor: Christine Portfors, Washington State University Decisions are customarily a result of the Reviewing Editor and the peer reviewers coming together and discussing their recommendations until a consensus is reached. When revisions are invited, a fact-based synthesis statement explaining their decision and outlining what is needed to prepare a revision will be listed below. The following reviewer(s) agreed to reveal their identity: Giulio Piperno.

The reviewers agree that this study provides important results to the field. Overall, this work represents a valid progress in the field, deepening our knowledge over autonoetic awareness and its association with the Bodily Self. The study uses an innovative use of VR and methodological approach to measure ANC.

Specific comments:

The introduction effectively frames the research topic, providing readers with clear definitions of the main experimental variables and the current state of the literature. However, several considerations should be addressed:

1. Rationale for Threat experience: Despite mentioning the role of emotional experience, it is unclear why the authors decided to measure SoA and SoO after a threatening experience. The description of the threat experience occurred unexpected when reading the following paragraph. Therefore, additional explanation should be provided to clarify and introduce this aspect.

2. Experimental Conditions: The authors present three experimental conditions to observe the association between SoA and SoO: one with both preserved, one with altered SoA, and the last with both altered SoA and SoO. However, it is unclear why the authors did not include a condition where only SoO was manipulated. Including such a condition would have allowed for an independent exploration of the roles of SoO and SoA.

3. Experimental Hypothesis: If authors had specific hypothesis on the role of SoO and SoA over ANC, they should state them more clearly. If not, it should be underlining the explorative nature of their analysis, an aspect to also highlight in the discussion section. Also, it should be specified whether they had different hypotheses specific to the subjective SoA rating and/or the experimental manipulation.

Methods

Authors did not mention how they estimated the sample size. If they did not run a priori power analysis, it could be useful to make a post-hoc estimation of the statistical power. Mixed models are a valid approach to explore the research question described by the authors, as they can account for both fixed and random effects. However, some clarifications are needed.

The authors initially analyzed the efficacy of the manipulation on SoA ratings, demonstrating how different experimental conditions (SYNCH1PP, ASYNCH1PP, &ASYNCH3PP) are associated with changes in SoA ratings. They then created another model incorporating both experimental conditions and SoA ratings. This raises two main concerns.

1) Collinearity: The model that includes both the experimental conditions and SoA ratings, along with their interaction, may suffer from a high degree of collinearity. The authors should at least provide the Variance Inflation Factor (VIF) to evaluate the feasibility of their statistical model. High VIF values would indicate problematic collinearity that needs to be addressed.

2) Interpretability of Results: The authors should clarify whether their effects are considered within-subject (i.e., the more a person experiences SoA at a given moment, the stronger the reliving of that moment) or between-subject (i.e., the more a person tends to experience SoA under normal conditions, the stronger their ANC). By examining the effects of the experimental manipulation, both in the graphs and statistical tables provided by the authors, it appears that there is a main effect on mean ANC (estimate = 2.8, p = 0.004), with higher mean scores in the condition with the greatest expected reduction in SoA and SoO (ASYNCH3PP). Conversely, in normal conditions (SoA and SoO preserved), the authors found a positive association between SoA ratings and ANC. This suggests that within-subject and between-subject effects may follow different patterns. This aspect should be considered and discussed in the results and discussion sections.

Figures Another relevant point is the understanding of the experimental manipulation and procedure. The authors should provide images of the three different conditions to facilitate the comprehension of their manipulation. Additionally, providing an image that outlines all the steps of the experiment would significantly enhance the reader's understanding.

Discussion

The discussion section is clear and well-structured. However, a few points could be considered to enhance its content:

1. Clarify Positive Association between SoA and ANC: The discussion should provide a clearer explanation of why there is a positive association between Sense of Agency (SoA) and Autonoetic Consciousness (ANC) only in the preserved condition. This could include a more detailed analysis of how the preserved condition supports the re-experiencing of events and the cognitive mechanisms that facilitate this association. Additionally, the results regarding the effect of ANC given by the experimental manipulation should be further elaborated.

2. Deepen Discussion on Brain Networks/Mechanisms: The authors briefly mention neural networks in the discussion. It would be beneficial to deepen this aspect by proposing specific neurocognitive mechanisms linking ANC and SoA. This could involve discussing relevant brain networks and how their interaction may support the integration of SoA and ANC. Providing explanation to existing neuroimaging studies that support these mechanisms would strengthen this discussion.

3. Implications for the Clinical Domain: The results could have significant implications for the clinical domain. Considering the role of imagery techniques and the re-experiencing of events in various therapeutic approaches, it would be useful to highlight how the role of SoA could be a relevant aspect to consider. For instance, discussing how enhancing SoA might improve the efficacy of therapeutic interventions that rely on vivid re-experiencing, such as imagery rescripting, could provide valuable insights. Emphasizing these clinical applications can underline the practical significance of the study's findings.

4. SoO and ANC: The absence of a condition where only Sense of Ownership (SoO) was manipulated means that this study cannot draw strong conclusions about the relationship between SoO and ANC. Moreover, the threat experience might have specific effects on SoO experienced by the participants (e.g., body dissociation as a defense mechanism). This should be discussed as a limitation of the current study, as SoO may indeed be similarly associated with ANC.

By addressing these points, the discussion can provide a more precise summary of the study's results and their broader implications.

## Author Response

Dear Dr. Portfors, We are happy to resubmit a revised version of our manuscript entitled "Sense of agency during encoding predicts subjective reliving" (eN-NWR-0256-24R1).

We thank you for the positive evaluation of our manuscript. Below, we provide a comprehensive point-by-point response (in blue) addressing each comment raised. All changes in the revised manuscript are indicated in bold and are underlined.

- Synthesis Statement for Author (Required):

1. The authors have provided a well-argued explanation for their choice to measure SoA and SoO following a threatening experience. However, I would suggest incorporating the references you used in your response to reviewers into the main text. This will help readers understand why threat ratings are an appropriate and valuable measure in your study. Additionally, it would be beneficial to mention in the discussion that the ratings of threat and SoO displayed a similar pattern, which would underscore the effectiveness of your experimental manipulation.

We have added the references in the main text, in the method section (page 7, lines 145-150):

Revised manuscript: "The addition of a threat allowed us to gauge SoO with a second more implicit question ( "I was afraid to be hurt by the knife"), to avoid potential floor effects for the main SoO question ("I felt that the virtual body was mine") (i.e., potential floor effect, because the visual aspects of the avatar did not match with those of the participants such as color, size, clothes, etc; Armel and Ramachandran, 2003; González-Franco et al., 2014; Kilteni et al., 2012; Ma and Hommel, 2015; Moon et al., 2022)." In addition, we have added some specification in the discussion section (page 18, lines 422-435):

Revised manuscript: "Although SoA and SoO were both systematically modulated across our conditions, we did not observe SoO-ANC coupling. We note that our experimental sensorimotor conditions aimed at modulating SoA, for which we observed significant reduction in both conditions with visuomotor and perspectival mismatch. Concerning SoO, we observed the same reduction of ratings in the strong visuomotor and perspectival mismatch condition (ASYNCH3PP) for both our explicit SoO ratings and our implicit measure of SoO (threat ratings), supporting the successful experimentally-induced decrease of bodily self-consciousness across our conditions with visuomotor and perspectival mismatch. Although Iriye and Ehrsson, (2022) showed that SoO, as manipulated by visuo-tactile stimulation during encoding, was positively related with increased vividness and emotional intensity in ANC ratings, collected one week later, several reasons may account for the difference between these and our results. First, the threat to the virtual body observed before the different ratings of bodily self-consciousness might have affected the SoO of participants and therefore hindered the potential SoO-ANC coupling not observed in our study." 2. The authors have clearly explained that the decision not to include a condition where only SoO was manipulated was driven by practical constraints rather than theoretical considerations. While this rationale is understandable, I suggest that you explicitly mention this limitation in the discussion section of your paper. Acknowledging this limitation and suggesting that future studies are needed to directly explore the independent roles of SoA and SoO would strengthen the overall discussion and provide valuable context for readers.

We have added this point to the limitation section of the discussion (page 20, lines 498-508):

Revised manuscript: "This study was not aimed at disentangling distinct effects of SoA versus SoO in ANC, but rather provide empirical evidence to show that ANC depends on sensorimotor bodily processes during encoding and its related subjective state of bodily self-consciousness. It would have been interesting to also test the effect of conditions in which only the SoO (and not the SoA) was altered, however, this would have significantly increased the already long experiment duration Accordingly, we employed a gradient, from SYNCH1PP to ASYNCH1PP to ASYNCH3PP, for which we expected a gradual manipulation of the SoA. This was sufficient to test our main research questions, while minimizing experiment duration, tiredness of participants, and task difficulty. Future studies should be designed to disentangle potentially distinct effects of subcomponent of bodily self-consciousness (i.e. SoA vs SoO) and their potential differential impact on ANC." 3. The authors have appropriately addressed this point, explaining that they did not have specific hypotheses regarding SoA versus SoO and that the subjective rating analyses were exploratory in nature. I would suggest that you further emphasize this last point by highlighting, in the introduction, that your study is among the first to consider the role of subjective ratings in this context. To this aim, it might be beneficial to include a similar version of the paragraph used in the response to the reviewers: "[..] most of the previous studies investigating the association of bodily self-consciousness and ANC did not measure the subjective state linked with the experimental manipulation of bodily self-consciousness (e.g., Bréchet et al., 2020, 2019; Gauthier et al., 2020). Moreover, the studies that did measure subjective ratings did not systematically investigate the link between these ratings during encoding and the later acquired subjective ANC evaluations during the reliving of the encoded scenes (e.g., Bergouignan et al., 2014; Iriye and Ehrsson, 2022)." Incorporating this information will help to underline the novelty of your approach and clarify the exploratory nature of your analysis, which will provide readers with valuable context.

We have added the part of our answer in the previous rebuttal letter to the introduction (pages 3-4, lines 56-72):

Revised manuscript: "Recent research developed several methods to modulate the SoA and SoO experimentally, by using virtual reality (VR) and exposing participants to different visuotactile or visuomotor stimulations (i.e., Fourneret and Jeannerod, 1998; Frank et al., 2001; Kannape et al., 2010; Kannape and Blanke, 2013; Salomon et al., 2022) or by changing the perspective from where the body of the participant and the virtual scene is seen (first-person perspective: 1PP; or third-person perspective: 3PP; Blanke et al., 2015; Ehrsson, 2012, 2007; Lenggenhager et al., 2014). However, only a few studies investigated the association between bodily self-consciousness and ANC, and most of them did not measure the subjective state linked with the experimental manipulation of bodily self-consciousness (e.g., Bréchet et al., 2020, 2019; Gauthier et al., 2020). To date only one study explored whether the experimental manipulation of the subjective SoO during encoding modulates the later subjective reliving of the encoded events (ANC). Iriye &Ehrsson (2022) observed that an altered SoO through visuo-tactile stimulation applied during the encoding period was associated with a decrease of some aspects of ANC, such as emotional intensity. However, the studies that did measure subjective ratings did not systematically investigate the link between these ratings during encoding and the later acquired subjective ANC (e.g., Bergouignan et al., 2014; Iriye and Ehrsson, 2022)." 4. The authors have appropriately addressed the previous concern about collinearity by conducting a VIF analysis. I recommend including the results of the VIF analysis in the manuscript. This addition would enhance the transparency of your statistical approach and provide readers with a clearer understanding of the robustness of your model We have added the description of the VIF analysis in the results section (page 12, lines 276-289) Revised manuscript: "Moreover, we found that depending on the encoding condition, the SoA was related to the ANC score as tested with a linear mixed model: there was a significant interaction between SYNCH1PP and ASYNCH3PP (with SoA estimate = -4.34, t = -2.99, p = 0.003, Figure 2B, Extended Data Figure 2-5), but not when comparing SYNCH1PP with ASYNCH1PP (estimate = -2.27, t = -1.58, p = 0.12). To investigate whether this interaction may have been due to collinearity in our linear mixed model, we calculated the variance inflation factor (VIF) for the model without interaction (ANC ~ Conditions + SoA + Experiment + random effect of participant) and found a VIF of 1 for each covariate (Conditions: 1.06, SoA:1.08, Experiment 1.01). We also quantified the VIF for the model with the interactions (SoA: 1.25, Experiment 1.01, Conditions: 6.1, Conditions:SoA: 5.9). As we found a VIF lower than 10 but higher than 5, our model indicates moderate collinearity and thus does not reach a level that would compromise the validity of our findings. Post-hoc power analysis revealed that the interaction had a statistical power of 71% (confidence interval were between 61.07 and 79.94)." 5. The authors have added a figure that indeed makes the experimental paradigm much easier to grasp. I have a couple of minor considerations:

1) The label for the ASYNCH1PP condition is somewhat hard to read due to the low contrast with the light blue background. I recommend adjusting the colors to improve readability.

2) The "threat" images are still missing from the figure. Including it, even in the supplementary materials, would be appropriate and helpful for a complete understanding of the experimental manipulation.

We thank the Reviewers for these comments. We have adjusted the colors to improve the readability of the ASYNCH1PP label, and have added a depiction of the threat in the figure. We have adapted the legend accordingly.

Figure 1 : Experimental design : Participants encoded three scene corresponding to three different conditions (left panel). One scene was encoded under preserved visuomotor and perspectival congruency (SYNCH1PP, dark blue), while another was encoded under visuomotor incongruency with preserved perspective (ASYNCH1PP, light blue). The last scene was encoded under visuomotor and perspectival incongruency (ASYNCH3PP, grey). The association between scene and condition was pseudorandomized between participants. After the encoding session, participants were immersed in a fourth outdoor scene (middle panel) in which they experienced the three conditions (SYNCH1PP, ASYNCH1PP and ASYNCH3PP). After performing the same movements as during encoding, they observed a virtual knife arriving in the virtual avatar (middle panel, colored circle). They were asked to rate their level of sense of agency (SoA), sense of ownership (SoO), whether they were afraid to be hurt by the knife (implicit SoO, threat ratings) and answers some control questions to evaluate their bodily self-consciousness. One week after the encoding session, participants were called back to answer an autonoetic consciousness questionnaire for each scene (and therefore each encoding condition).

6. The authors have appropriately expanded the discussion offering a more detailed explanation of the possible mechanisms behind the significant interaction. I noticed a small typo in the newly added text: the phrase "of brain regions" is repeated twice. I suggest correcting this to ensure the text is clear and error-free.

We thank the Reviewers for this comment and we have removed the typos. The corrected sentence is now as follows (page 17, lines 409-412):

Revised manuscript: "Our results indicate that the hippocampus may also index the reactivation of brain regions involved in bodily self-consciousness (Park and Blanke, 2019) during the re-experience of an event, especially when this event was encoded under preserved visuomotor and perspectival congruency."
