# Supplementary material for: Sense of Agency during Encoding Predicts Subjective Reliving
Source: eNeuro. 2024 Oct 10;11(10):ENEURO.0256-24.2024. doi: 10.1523/ENEURO.0256-24.2024 (PMC11613308; doi:10.1523/ENEURO.0256-24.2024)
Supplement: Figure 2-1 — Sense of agency. Sense of agency ∼ Conditions + Experiment + random(Participants). Download Figure 2-1, DOCX file. [file eneuro-11-ENEURO.0256-24.2024-s005.docx]

|  | estimate | t | p |
| --- | --- | --- | --- |
| (Intercept) | 0.690 *** | 13.575 | < 0.001** |
| Conditions ASYNCH1PP | -0.058 | -2.866 | 0.003 ** |
| Conditions ASYNCH3PP | -0.083 | -4.28 | < 0.001 *** |
| Experiment 1 | -0.03 | -0.48 | 0.63 |
| Experiment 2 | -0.092 | -1.38 | 0.17 |

Figure 2 - 1: Sense of agency. Sense of agency ~ Conditions +Experiment + random(Participants)
