# Supplementary material for: Sense of Agency during Encoding Predicts Subjective Reliving
Source: eNeuro. 2024 Oct 10;11(10):ENEURO.0256-24.2024. doi: 10.1523/ENEURO.0256-24.2024 (PMC11613308; doi:10.1523/ENEURO.0256-24.2024)
Supplement: Figure 2-2 — Sense of ownership. Sense of ownership ∼ Conditions + Experiment + random(Participants). Download Figure 2-2, DOCX file. [file eneuro-11-ENEURO.0256-24.2024-s004.docx]

|  | estimate | t | p |
| --- | --- | --- | --- |
| (Intercept) | 0.568 | 10.874 | < 0.001** |
| Conditions ASYNCH1PP | -0.003 | -0.12 | 0.9 |
| Conditions ASYNCH3PP | -0.12 | -4.62 | < 0.001 *** |
| Experiment 1 | -0.09 | -1.19 | 0.24 |
| Experiment 2 | -0.15 | -2.14 | 0.036 * |

Figure 2 - 2: Sense of ownership. Sense of ownership ~ Conditions + Experiment + random(Participants)
