# Supplementary material for: Sense of Agency during Encoding Predicts Subjective Reliving
Source: eNeuro. 2024 Oct 10;11(10):ENEURO.0256-24.2024. doi: 10.1523/ENEURO.0256-24.2024 (PMC11613308; doi:10.1523/ENEURO.0256-24.2024)
Supplement: Figure 2-3 — Threat. Threat ∼ Conditions + Experiment + random(Participants). Download Figure 2-3, DOCX file. [file eneuro-11-ENEURO.0256-24.2024-s003.docx]

|  | estimate | t | p |
| --- | --- | --- | --- |
| (Intercept) | 0.239 | 5.15 | < 0.001** |
| Conditions ASYNCH1PP | -0.02 | -0.75 | 0.45 |
| Conditions ASYNCH3PP | -0.117 | -3.92 | < 0.001 *** |
| Experiment 1 | 0.02 | 0.37 | 0.71 |
| Experiment 2 | 0.056 | 0.92 | 0.36 |

Figure 2 - 3: Threat. Threat ~ Conditions + Experiment + random(Participants)
