# Supplementary material for: Sense of Agency during Encoding Predicts Subjective Reliving
Source: eNeuro. 2024 Oct 10;11(10):ENEURO.0256-24.2024. doi: 10.1523/ENEURO.0256-24.2024 (PMC11613308; doi:10.1523/ENEURO.0256-24.2024)
Supplement: Figure 2-4 — Control.Control ∼ Conditions + Experiment + random(Participants). Download Figure 2-4, DOCX file. [file eneuro-11-ENEURO.0256-24.2024-s002.docx]

|  | estimate | t | p |
| --- | --- | --- | --- |
| (Intercept) | 0.17 | 6.16 | < 0.001** |
| Conditions ASYNCH1PP | 0.006 | 0. 59 | 0.55 |
| Conditions ASYNCH3PP | -0.019 | -1.83 | 0.06 |
| Experiment 1 | -0.098 | -2.55 | 0.013 * |
| Experiment 2 | -0.026 | -0.69 | 0.49 |

***Figure 2 - 4: Control.****Control ~ Conditions + Experiment + random(Participants)*
