# Supplementary material for: Sense of Agency during Encoding Predicts Subjective Reliving
Source: eNeuro. 2024 Oct 10;11(10):ENEURO.0256-24.2024. doi: 10.1523/ENEURO.0256-24.2024 (PMC11613308; doi:10.1523/ENEURO.0256-24.2024)
Supplement: Figure 2-5 — Autonoetic consciousness explained by SoA and Conditions. ANC ∼ Conditions * SoA + Experiment + random(Participants). Download Figure 2-5, DOCX file. [file eneuro-11-ENEURO.0256-24.2024-s009.docx]

|  | estimate | t | p |
| --- | --- | --- | --- |
| (Intercept) | 14.07 | 12.6 | < 0.001** |
| Conditions ASYNCH1PP | 1.76 | 1.82 | 0.07 |
| Conditions ASYNCH3PP | 2.8 | 2.9 | 0.004 *** |
| Sense of Agency | 1.96 | 1.36 | 0.17 |
| Experiment 1 | -0.57 | -0.68 | 0.5 |
| Experiment 2 | -0.76 | -0.92 | 0.36 |
| Conditions ASYNCH1PP × Sense of Agency | -2.28 | -1.58 | 0.12 |
| Conditions ASYNCH3PP × Sense of Agency | -4.33 | -2.99 | 0.0033 ** |

Figure 2 - 5: Autonoetic consciousness explained by SoA and Conditions. ANC ~ Conditions * SoA + Experiment +random(Participants)
