# Supplementary material for: Sense of Agency during Encoding Predicts Subjective Reliving
Source: eNeuro. 2024 Oct 10;11(10):ENEURO.0256-24.2024. doi: 10.1523/ENEURO.0256-24.2024 (PMC11613308; doi:10.1523/ENEURO.0256-24.2024)
Supplement: Figure 2-6 — Autonoetic consciousness explained by Control and Conditions. ANC ∼ Conditions * Control + Experiment + random(Participants). Download Figure 2-6, DOCX file. [file eneuro-11-ENEURO.0256-24.2024-s008.docx]

|  | estimate | t | p |
| --- | --- | --- | --- |
| (Intercept) | 14.63 | 16.18 | < 0.001** |
| Conditions ASYNCH1PP | 0.51 | 0.7 | 0.49 |
| Conditions ASYNCH3PP | 0.99 | 1.39 | 0.17 |
| Ownership | 0.98 | 0.78 | 0.43 |
| Experiment 1 | -0.55 | -0.66 | 0.51 |
| Experiment 2 | -0.66 | -0.8 | 0.42 |
| Conditions ASYNCH1PP × Ownership | -0.42 | -0.32 | 0.75 |
| Conditions ASYNCH3PP × Ownership | -1.86 | -1.33 | 0.19 |

***Figure 2 - 6: Autonoetic consciousness explained by SoO and Conditions.*** *ANC ~ Conditions * Ownership + Experiment + random(Participants)*
