# Supplementary material for: Sense of Agency during Encoding Predicts Subjective Reliving
Source: eNeuro. 2024 Oct 10;11(10):ENEURO.0256-24.2024. doi: 10.1523/ENEURO.0256-24.2024 (PMC11613308; doi:10.1523/ENEURO.0256-24.2024)
Supplement: Figure 2-7 — Autonoetic consciousness explained by Threat and Conditions. ANC ∼ Conditions * Threat + Experiment + random(Participants). Download Figure 2-7, DOCX file. [file eneuro-11-ENEURO.0256-24.2024-s007.docx]

|  | estimate | t | p |
| --- | --- | --- | --- |
| (Intercept) | 15.06 | 22.33 | < 0.001** |
| Conditions ASYNCH1PP | 0.8 | 1.63 | 0.1 |
| Conditions ASYNCH3PP | 0.6 | 1.24 | 0.22 |
| Threat | 0.5 | 0.47 | 0.64 |
| Experiment 1 | -0.67 | -0.8 | 0.43 |
| Experiment 2 | -0.73 | -9 | 0.37 |
| Conditions ASYNCH1PP × Threat | -2 | -1.5 | 0.14 |
| Conditions ASYNCH3PP ×Threat | -2.32 | -1.39 | 0.17 |

Figure 2 - 7: Autonoetic consciousness explained by Threat and Conditions. ANC ~ Conditions * Threat + Experiment + random(Participants)
