# Supplementary material for: Sense of Agency during Encoding Predicts Subjective Reliving
Source: eNeuro. 2024 Oct 10;11(10):ENEURO.0256-24.2024. doi: 10.1523/ENEURO.0256-24.2024 (PMC11613308; doi:10.1523/ENEURO.0256-24.2024)
Supplement: Figure 2-8 — Autonoetic consciousness explained by SoO and Conditions. ANC ∼ Conditions * Ownership + Experiment + random(Participants). Download Figure 2-8, DOCX file. [file eneuro-11-ENEURO.0256-24.2024-s006.docx]

|  | estimate | t | p |
| --- | --- | --- | --- |
| (Intercept) | 15.12 | 20.52 | < 0.001** |
| Conditions ASYNCH1PP | 0.37 | 0.8 | 0.42 |
| Conditions ASYNCH3PP | 0.53 | 1.11 | 0.27 |
| Control | 0.6 | 0.25 | 0.8 |
| Experiment 1 | -0.66 | -0.76 | 0.45 |
| Experiment 2 | -0.76 | -0.92 | 0.36 |
| Conditions ASYNCH1PP ×Control | -0.6 | -0.26 | 0.79 |
| Conditions ASYNCH3PP ×Control | -3.01 | -1.1 | 0.27 |

Figure 2 - 8: Autonoetic consciousness explained by Control and Conditions. ANC ~ Conditions * Control + Experiment + random(Participants)
